# Topical and systemic GLP-1R agonist administration both rescue retinal ganglion cells in hypertensive glaucoma

**DOI:** 10.3389/fncel.2023.1156829

**Published:** 2023-06-09

**Authors:** Emily C. N. Lawrence, Michelle Guo, Turner D. Schwartz, Jie Wu, Jingwen Lu, Sergei Nikonov, Jacob K. Sterling, Qi N. Cui

**Affiliations:** Department of Ophthalmology, Scheie Eye Institute, University of Pennsylvania, Philadelphia, PA, United States

**Keywords:** glaucoma, neuroprotection, glucagon-like peptide-1 receptor agonist, retinal ganglion cell (RGC), optic nerve (ON), microglia, neuroinflammation, macrophage

## Abstract

Glaucomatous neurodegeneration, a blinding disease affecting millions worldwide, has a need for the exploration of new and effective therapies. Previously, the glucagon-like peptide-1 receptor (GLP-1R) agonist NLY01 was shown to reduce microglia/macrophage activation, rescuing retinal ganglion cells after IOP elevation in an animal model of glaucoma. GLP-1R agonist use is also associated with a reduced risk for glaucoma in patients with diabetes. In this study, we demonstrate that several commercially available GLP-1R agonists, administered either systemically or topically, hold protective potential in a mouse model of hypertensive glaucoma. Further, the resulting neuroprotection likely occurs through the same pathways previously shown for NLY01. This work contributes to a growing body of evidence suggesting that GLP-1R agonists represent a viable therapeutic option for glaucoma.

## Introduction

Glaucoma is a neurodegenerative disease characterized by retinal ganglion cell (RGC) death and optic nerve (ON) atrophy, causing progressive and permanent vision loss. As the leading cause of irreversible blindness worldwide, glaucoma is projected to affect 112 million people by 2040 (Tham et al., 2014). Currently, lowering intraocular pressure (IOP) is the only proven therapeutic option for glaucoma patients. However, a significant number of patients still experience disease progression despite appreciable IOP lowering (Quigley, 2019), underscoring the need for novel therapies for this disease.

Synthetic glucagon-like peptide-1 receptor (GLP-1R) agonists are a popular class of therapy for type 2 diabetes mellitus, which a number of studies have suggested may be a risk factor for glaucoma (Hayes et al., 2014; Zhou et al., 2014; Tella and Rendell, 2015; Zhao et al., 2015). In the central nervous system, GLP-1R activation initiates a signaling cascade that inhibits the release of proinflammatory cytokines and microglia transformation (Athauda and Foltynie, 2016). This pathway is one of the mechanisms through which GLP-1R agonists are thought to provide neuroprotection in multiple *in vitro* and animal models of Parkinson’s and Alzheimer’s neurodegeneration (Erbil et al., 2019).

Glucagon-like peptide-1 receptor agonists are peptide drugs developed as analogs of either human GLP-1 or exendin-4, a salivary protein from the Gila monster with ∼50% homology to human GLP-1 (Gentilella et al., 2019). All GLP-1R agonists elicit downstream effects by binding to the GLP-1 receptor, a transmembrane G-protein-coupled receptor that is expressed in the CNS as well as the pancreas and the gut (Athauda and Foltynie, 2016). Human GLP-1-based agents include liraglutide, dulaglutide, and semaglutide, whereas exenatide and lixisenatide are based on exendin-4 (Gentilella et al., 2019). Synthetic GLP-1R agonists are widely used to treat type 2 diabetes, in which the drugs demonstrate both peripheral and central effects (Hunter and Hölscher, 2012). Liraglutide and semaglutide were also recently approved for weight-loss in non-diabetic patients (Rubino et al., 2021; Deng et al., 2022).

In the central nervous system (CNS), GLP-1R activation initiates a signaling cascade that inhibits the release of proinflammatory cytokines and microglia transformation, both key contributors to Parkinson’s and Alzheimer’s pathogenesis (Athauda and Foltynie, 2016; Cui et al., 2022). Through this anti-inflammatory effect, GLP-1R agonists are protective in several *in vitro* and animal models of neurodegeneration (Bertilsson et al., 2008; Harkavyi et al., 2008; Kim et al., 2009; Li et al., 2009; Erbil et al., 2019). Specific to the eye, GLP-1R agonists have been found to be protective in experimental models of diabetic retinopathy by downregulating proinflammatory and apoptotic pathways, decreasing reactive astrogliosis, and preventing disruption of the blood retina barrier (Fan et al., 2014; Hernández et al., 2016).

Using the microbead-induced mouse model of hypertensive glaucoma, we have previously shown that microglia/macrophage upregulate C1q, TNF-α, and IL-1α, three cytokines necessary and sufficient for neurotoxic astrogliosis within the retina, leading to RGC loss. In the same hypertensive glaucoma model, we showed that systemic administration of the synthetic GLP-1R agonist NLY01 reduced the production of these same proinflammatory cytokines by microglia/macrophage, prevented reactive astrocyte transformation, and rescued RGCs (Sterling et al., 2020). These findings motivated our hypothesis that GLP-1R agonists protect against RGC death by decreasing monocyte infiltration and microglia/macrophage activation. Further, using retrospective insurance claims data, we found that treatment with existing U.S. Food and Drug Administration (USFDA)-approved GLP-1R agonists, including exenatide, liraglutide, albiglutide, dulaglutide, semaglutide, and lixisenatide, was associated with a 44% risk reduction for a new diagnosis of glaucoma or glaucoma suspect in diabetic patients (hazard ratio 0.56, 95% CI: 0.36 to 0.89, *p* = 0.01; Sterling et al., 2021). By removing “glaucoma suspect” from the outcome definition, an even stronger protective association for incident glaucoma was found following GLP-1R agonist exposure (hazard ratio 0.08, 95% CI: 0.02 to 0.42, *p* = 0.003). These findings suggest that commercially available GLP-1R agonists may also be an effective therapy for patients at risk for glaucoma.

The purpose of this study is to determine if commercially available GLP-1R agonists, administered either systemically or topically, are protective in a mouse model of hypertensive glaucoma, and whether any protection that results occur through the same pathways we have previously shown with NLY01. The GLP-1R agonists liraglutide and lixisenatide were selected because topical formulations of both as well as systemic administration of liraglutide demonstrated effectiveness against retinal neurodegeneration in a mouse model of diabetes (Hernández et al., 2016). Eyes that received systemic NLY01 were also included for RGC functional testing and optic nerve analyses as these were not previously done (Sterling et al., 2020).

## Materials and methods

The Microbead Occlusion Model of glaucoma was used to induce ocular hypertension as previously described (Sterling et al., 2020). Briefly, 3-month-old C57BL/6J mice (Jackson Laboratory, Bar Harbor, ME, USA) received bilateral injections of approximately 1.5 μl of sterile 4.5 μm-diameter magnetic microbeads [1.6 × 10^6^ beads/μl of balanced salt solution (BSS); Dynabeads M-450 Epoxy, Thermo Fisher Scientific, Waltham, MA, USA] into the anterior chamber. Control mice received bilateral sterile BSS injections of the same volume. IOPs were measured in awake animals between 8 and 11 a.m. local time. IOPs were measured immediately prior to the first injection, then at regular intervals post-injection using the Icare^®^ TONOLAB tonometer (Icare TONOVET, Vantaa, Finland). An average of 3 measurements per eye was used.

Treatment groups received either topical or systemic GLP-1R agonists, initiated 3 days after ocular injections. In the topical group, either lixisenatide [20 μg/kg/day, in filtered sterilized normal saline (NS)] or liraglutide (400 μg/kg/day, in filtered sterilized NS) was administered twice daily to the ocular surface of one eye using a micropipette, while the fellow eye received an equivalent volume of NS as previously described (Shu et al., 2019). Each animal was weighed at the beginning of the study and dosing volume was determined based on concentration, rounded to the nearest microliter (averaging 5 μl/day). That dose was used for the duration of the study.

In the systemic group, animals received either liraglutide (400 μg/kg) once daily or NLY01 (5 mg/kg) twice weekly administered subcutaneous, while control animals received subcutaneous PBS injections of the same volume and frequency. For a complete list of all treatment conditions, refer to Table 1.

**TABLE 1:** Treatment conditions for both study groups.

Topical		Systemic
OD	OS	OU
BSS/Lixisenatide	BSS/NS	BSS/PBS
BSS/Liraglutide	BSS/NS	Bead/PBS
Bead/Lixisenatide	Bead/NS	BSS/NLY01
Bead/Liraglutide	Bead/NS	Bead/NLY01
		BSS/Liraglutide
		Bead/Liraglutide

Format: (intraocular injection/treatment condition). OD, right eye; OS, left eye; OU, both eyes. In the topical groups, OD was compared to OS within the same animal. In the systemic groups, both eyes of a treated animal were compared to both eyes of an untreated animal.

Retinal ganglion cell and microglia counting and immunolabeling of flat-mounted retinas were performed as previously described with minor modification noted below (Sterling et al., 2020). Briefly, eyes were enucleated immediately after sacrifice and fixed for 30 min in 4% paraformaldehyde. The neurosensory retinas were isolated, flattened through the creation of four cuts, and mounted on glass slides. Following serial washes with 0.5% Triton X-100 in PBS (TPBS) and 15-min permeabilization through freeze/thaw at −80°C, retinas were incubated overnight at 4°C with guinea pig anti-RBPMS (ABN1376, MilliporeSigma, Burlington, MA, USA) diluted 1:500 in a blocking buffer (2% bovine serum albumin and 2% Triton X-100 in PBS) and rabbit anti-Iba1 (ab178846, Abcam, Cambridge, UK) diluted 1:1000 in the same blocking buffer. The following day, retinas were washed in TPBS and incubated for 3 h at RT with Alexa Fluor 488 donkey anti-rabbit secondary antibody (A21206, Invitrogen, Waltham, MA, USA) and Cy3 goat anti-guinea pig secondary antibody (ab102370, Abcam), diluted 1:1000 and 1:500, respectively, in blocking buffer. Finally, retinas were washed, and cover slipped with Vectashield mounting medium plus DAPI for fluorescence (Vector Laboratories Inc., Burlingame, CA, USA). For each retina, 12 standardized photomicrographs at 1/6, 3/6, and 5/6 distance from the center of the retina were taken in four quadrants at 40X magnification with the ganglion cell layer in focus. A masked counter then quantified the number of RBPMS-positive RGC and Iba1-positive microglia/macrophage somas in each image using the FIJI “cell-counter” plug-in. Each counted cell was verified by observing DAPI nuclear staining in addition to RBPMS or Iba1 signal for RGCs and microglia/macrophages, respectively. A cell was counted only if greater than 50% of the soma was within the imaged frame, exclusive of cellular projections. One retina each from the BSS/topical lixisenatide group, the bead/topical lixisenatide group, and the bead/topical liraglutide group was irretrievably damaged during dissection. A pair of retinas in the BSS/topical liraglutide group was excluded from myeloid cell counts due to insufficient Iba1 staining.

Optic nerves (ONs) were bisected at the level of the nerve head and fixed in 2.5% glutaraldehyde with 2.0% paraformaldehyde in 0.1 M sodium cacodylate buffer, pH 7.4. RGC axons were labeled and counted as previously described with minor modification noted below (Cui et al., 2020). Briefly, ONs were incubated in 2% osmium tetroxide and dehydrated in graded ethanol immersions. Following embedding in epoxy resin Embed 812 (Electron Microscopy Sciences, Hatfield, PA, USA), 0.75 μm thick cross sections were generated from a section of the nerve 1.5 mm posterior to the globe and stained with 1% toluidine blue. For each ON, a composite image of the entire cross section was created by obtaining and stitching together standard photomicrographs at 100X magnification using the Axio Observer 7 microscope (Carl Zeiss Inc., White Plains, NY, USA). Axon counts were obtained by a masked operator using the Axonet Image Analysis Algorithm plugin for FIJI (Ritch et al., 2020). An average of axon counts from 3 whole nerve cross sections per nerve was used. One ON from the systemic NLY01 group was excluded due to poor tissue processing resulting in impaired image quality.

Miltenyi Adult Brain Dissociation Kit (130-107-677, Miltenyi Biotec, Bergisch Gladbach, Germany) and MACS magnetic cell separation system was used to enrich retinal cellular populations as previously described (Sterling et al., 2020). Sequential isolation steps resulted in the following populations: (1) ACSA2 + (130-097-678, Miltenyi Biotec), (2) ACSA2- CD11b + (130-126-725, Miltenyi Biotec), and (3) ACSA2- CD11b-. RNA isolation followed by quantitative PCR (qPCR) was performed as previously described on the cellular population enriched for microglia and macrophages (ACSA2- CD11b +). qPCR (Applied Biosystems TaqMan, Thermo Fisher Scientific, Waltham, MA, USA) was performed in biological and technical triplicates on a sequence detection system to provide normalized expression values.

Microelectrode array (MEA) recordings were performed using modifications of previously described methods (Gaub et al., 2014; Song et al., 2018). Briefly, small patches of dark-adapted retinas were placed ganglion cell side down in the perforated MEA chamber mounted on the 1060i amplifier (Multi Channel Systems, Reutlingen, Germany), and gentle suction was applied to the retina to improve its contact with the electrodes. The chamber was perfused with oxygenated Ames’ solution maintained at 37°C. The retina was stimulated using calibrated flashes of 455 nm light. The data was acquired using a NI PCI-6071E DAQ board and custom software developed in LabView (National Instruments, Austin, TX, USA), and analyzed using custom MATLAB (MATLAB, Natick, MA, USA) based code. MEA-related figures were rendered using the export_fig utility (Altman, 2023).

## Results

**FIGURE 1:**
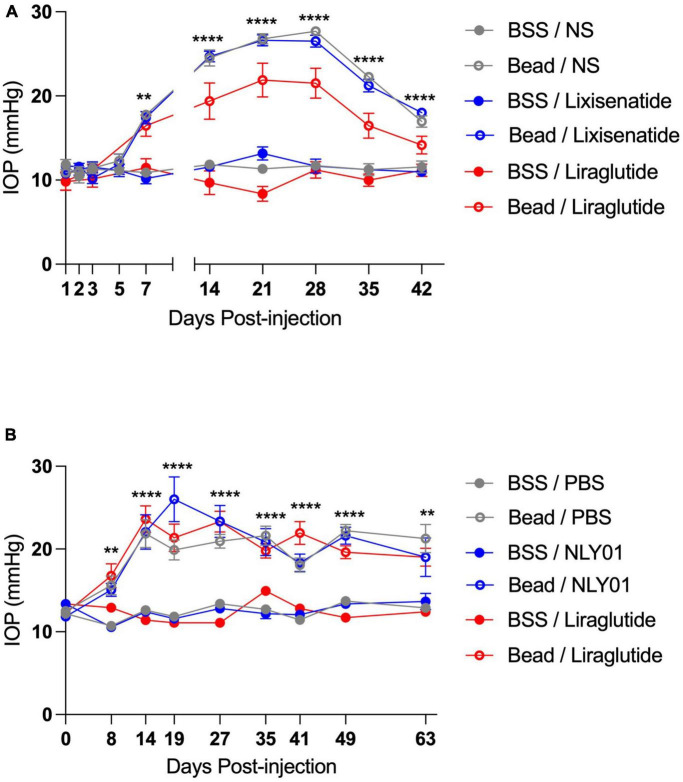
Injection of microbeads into the anterior chamber elevates IOP. C57BL6/J mice received either bilateral magnetic microbead (Bead) or balanced salt solution (BSS) injections into the anterior chamber, followed by either topical **(A)** or systemic **(B)** administration of GLP-1R agonists. Topical BSS on the fellow eye served as control for the topical group while subcutaneous PBS served as control for the systemic group. Bead-injected eyes had significantly elevated IOP compared to the BSS-injected eyes at all-time points after and including day 7 post-injection. All data are presented as mean ± SEM. ^**^p < 0.01 and ^****^p < 0.0001, one-way ANOVA with Tukey’s method compared between bead- and BSS-injected groups. Comparisons within bead-injected groups and within BSS-injected groups were not significantly different with the exception of topical Bead/Liraglutide compared to the other topical bead-injected groups on days 21, 28, 35, and 42 (*p* < 0.05 for all). Topical groups: *N* = 10. Systemic groups: *N* = 20.

Following ocular injections, IOP began to elevate around day 7 in the bead-injected eyes compared to BSS-injected eyes and remained elevated until end-point analyses at day 42 for the topical groups and day 63 for the systemic groups (*p* < 0.01 for all time-points between bead- and BSS-injected eyes including and after day 7; Figure 1). Interestingly, while IOPs were not significantly different between the majority of bead-inject groups, the topical liraglutide bead-injected group demonstrated significantly lower IOPs than the topical BSS and lixisenatide bead-injected groups at Days 21, 28, 35, and 42 (*p* < 0.05 for these time points).

**FIGURE 2:**
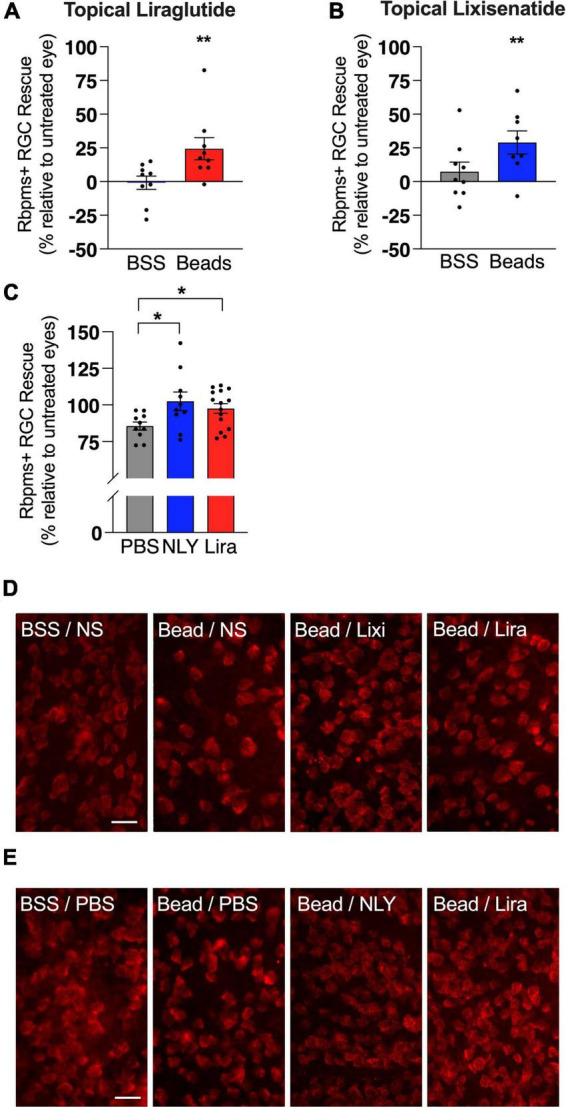
Topical and systemic GLP-1R agonists both rescue RGCs in hypertensive glaucoma. At 42 [topical group; **(A,B)**] and 63 days [systemic group; **(C)**] post-microbead injections, retina flat mounts were stained for the retinal ganglion cell (RGC) marker RBPMS. Percent RGC rescue, calculated against the untreated fellow eye in the topical group and against the average of the untreated eyes in the systemic group, was significant for all treatment conditions post-microbead-induced ocular hypertension. Representative images for the topical **(D)** and the systemic **(E)** group. Scale bar 50 μm. All data are presented as mean ± SEM. *p < 0.05, **p < 0.01, paired (topical) and unpaired (systemic) Student’s *t*-test, compared within bead-injected and BSS-injected groups.

**FIGURE 3:**
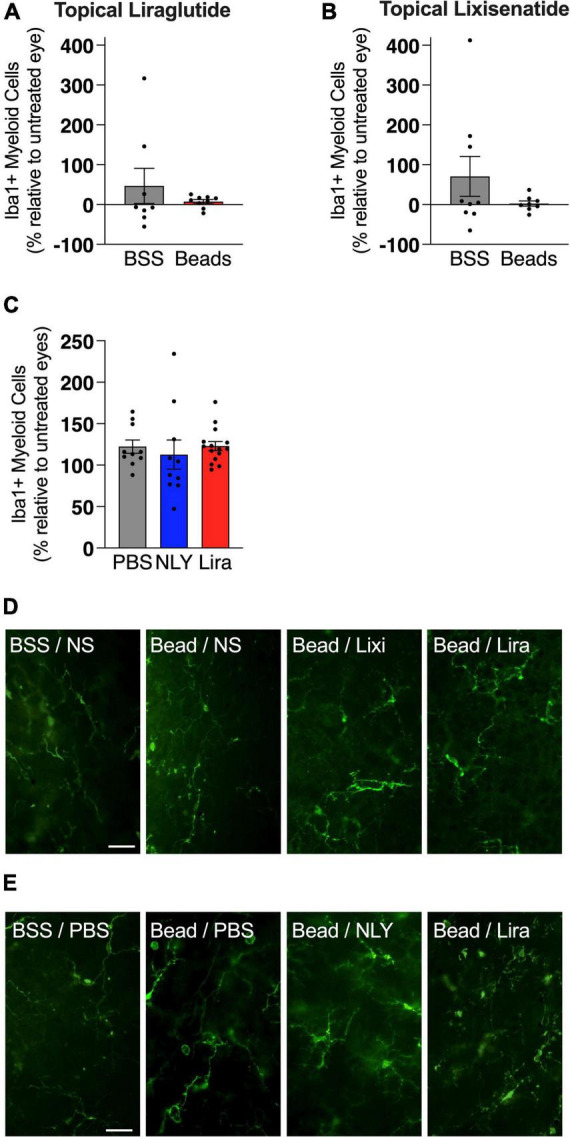
GLP-1R agonists do not affect the number of iba1 + cells in the retina. At 42 [topical group; **(A,B)**] and 63 [systemic group; **(C)**] days post-microbead injections, retina flat mounts were stained for the myeloid cell marker Iba1. Iba1 + cells, calculated as a percentage of the untreated fellow eye in the topical group and as a percentage of the average of untreated eyes in the systemic group, did not significantly differ based on treatment conditions. Representative images for the topical **(D)** and the systemic **(E)** group. Scale bar 50 μm. All data are presented as mean ± SEM. Paired (topical) and unpaired (systemic) Student’s *t*-test.

In the setting of ocular hypertension, topical liraglutide administration resulted in a 24% rescue of RGCs relative to the untreated, bead-injected fellow eye (*p* = 0.0048), while topical lixisenatide administration resulted in a 29% rescue (*p* = 0.0096; Figure 2). In comparison, systemic administration of NLY01 and liraglutide resulted in complete rescue of RGCs after microbead-induced IOP elevation (*p* = 0.0243 and 0.0170 relative to untreated, bead-injected eyes, respectively). The number of Iba1 + myeloid cells did not significantly differ between treatment and controls in either the topical or systemic group (Figure 3).

**FIGURE 4:**
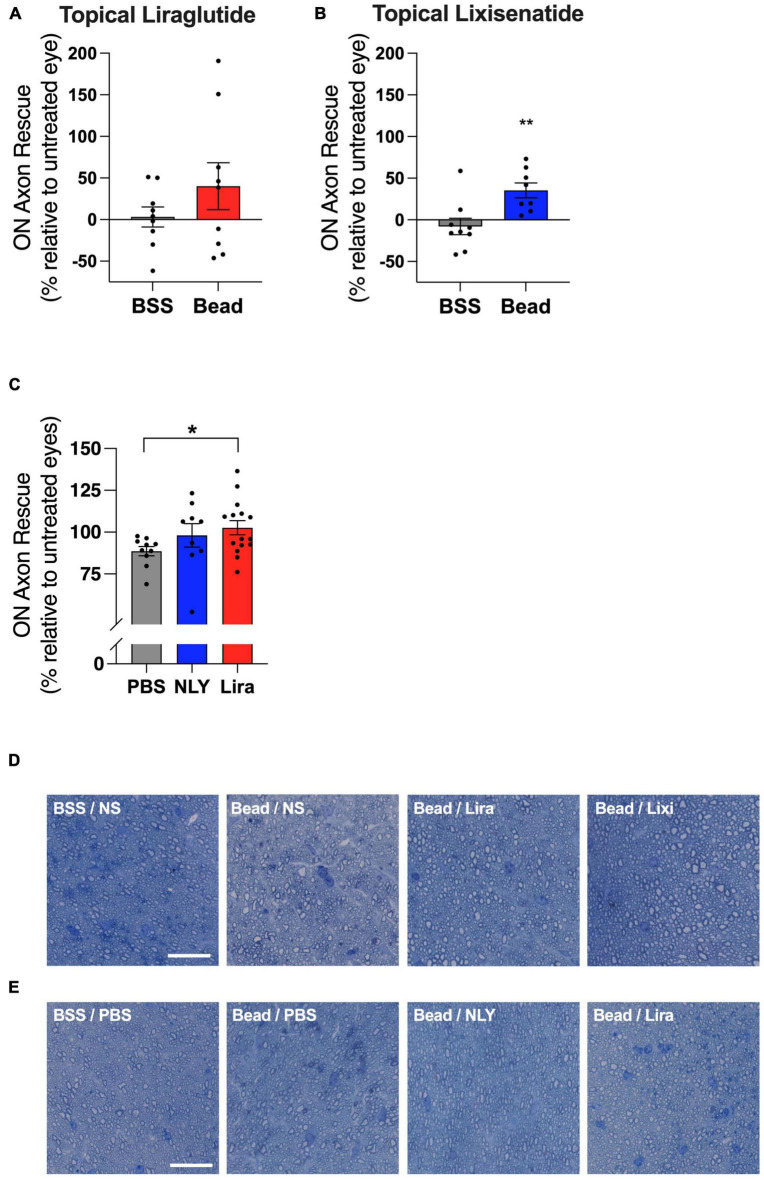
Topical and systemic GLP-1R agonists variably preserve optic nerve axons in hypertensive glaucoma. At 42 [topical group; **(A,B)**] and 63 [systemic group; **(C)**] days post-microbead injections, RGC axons were quantified from a cross section of the optic nerve posterior to the globe. Percent axon rescue, calculated against the untreated fellow eye in the topical group and against the average of the untreated eyes in the systemic group, was significant following treatment with topical lixisenatide **(B)** and systemic liraglutide **(C)**. Representative images (cropped to 75 um^2^) are shown for the topical **(D)** and the systemic **(E)** group. Scale bar 20 μm. All data are presented as mean ± SEM. *p < 0.05 and **p < 0.01, paired (topical) and unpaired (systemic) Student’s *t*-test.

In the setting of ocular hypertension, topical lixisenatide resulted in a 35% rescue of ON axons relative to the untreated, bead-injected fellow eye (*p* = 0.0060, Figure 4). Percent rescue of ON axons following topical liraglutide administration did not demonstrate significance (*p* = 0.2450). In the systemic group, liraglutide resulted in near complete rescue of ON axons relative to untreated, bead-injected eyes, while NLY01 demonstrated a trend towards improved axon survival that did not cross the threshold of significance relative to untreated, bead-injected eyes (*p* = 0.0214 and 0.2106, respectively).

**FIGURE 5:**
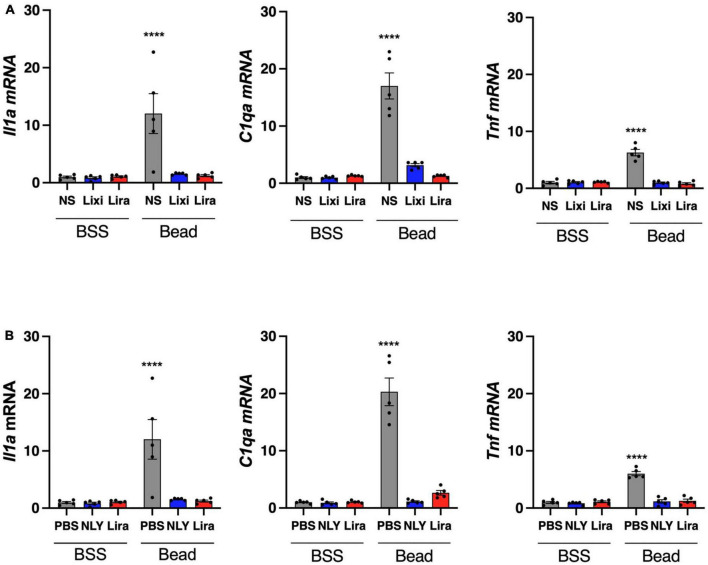
Topical and systemic GLP-1R agonists decrease pro-inflammatory cytokine expression in hypertensive glaucoma. At 42 [topical group; **(A)**] and 63 [systemic group; **(B)**] days post-microbead injections, a cellular population enriched for microglia/macrophage (CD11b +) were isolated from neurosensory retina. Expression of *Il1a*, *Tnf*, and *C1qa* mRNA decreased in both topic and systemic groups following treatment with GLP-1R agonists. All data are presented as mean ± SEM. ****p < 0.0001, paired (topical) and unpaired (systemic) Student’s *t*-test.

*Il1a*, *Tnf*, and *C1qa* mRNA were upregulated in microglia/macrophage-enriched cellular populations of bead-injected eyes without GLP-1R agonist administration (Figure 5). These markers of neuroinflammation were downregulated in all bead-injected eyes following treatment with both topical and systemic administration of GLP-1R agonists (*p* < 0.0001 for all groups). Results recapitulate our previous findings for systemic NLY01 administration (Sterling et al., 2020).

**FIGURE 6:**
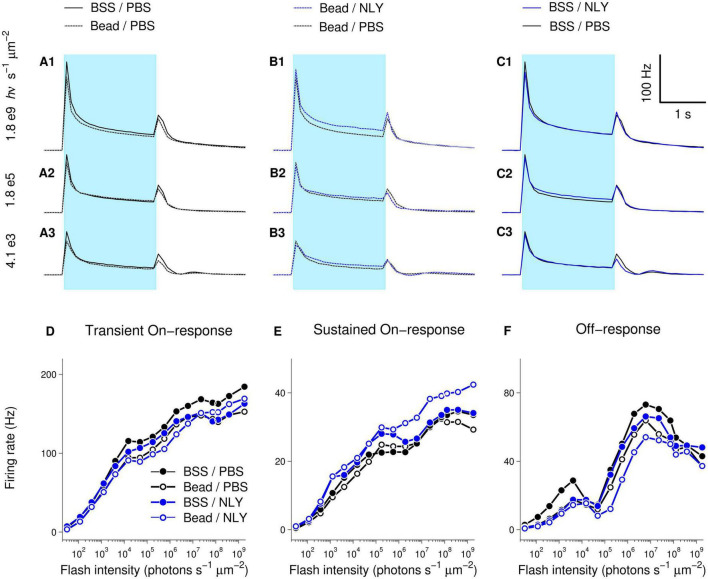
The GLP-1R agonist NLY01 improves assessment of RGC function in hypertensive glaucoma. At 63 days post-ocular injections, small patches of NLY01 treated and control retinas were stimulated with flashes of light for multi-electrode array recording. Group-averaged (per electrode per flash-averaged) retinal responses to flashes of three different intensities were calculated using 100 ms time bin, with each row corresponding to a particular intensity as indicated on the far left of graphs **(A–C)**. Blue bar = light stimulation. Amplitudes of transient On-[peak firing rate after flash onset minus baseline firing rate; **(D)**], sustained On-[firing rate before flash offset minus baseline firing rate; **(E)**], and Off-[peak firing rate after flash offset minus firing rate before flash offset; **(F)**] responses suggest better RGC function following NLY01 treatment that is most pronounced at higher flash intensities and for the sustained On-response. *N* = 11 for BSS/PBS, *N* = 10 for Bead/PBS; *N* = 4 for Bead/NLY01, *N* = 6 for BSS/NLY01.

Diminished response amplitudes observed in MEA recordings suggest decreased RGC function in untreated eyes with IOP elevation, which were particularly evident at brighter intensities (Bead/PBS compared to BSS/PBS, Figure 6). On-responses in the NLY01 treated group (Bead/NLY) were increased compared to responses in the untreated Bead/PBS group. This effect can be observed at the brighter intensities for the transient On-, and across the whole intensity range for the sustained On-responses, while Off-responses were diminished. Note that different vertical scales were used to plot measurements from different response types.

Examination of response-intensity curves from individual retinas had shown that two retinas in the Bead/PBS group were unusual in that they produced responses as large as those from the best responding retinas in the control BSS/PBS group. To identify retinas with responses most deviant from group-averages, we calculated 99% confidence intervals for the BSS/PBS and Bead/PBS groups at each tested intensity (retina counts for the other two groups were too low for meaningful confidence intervals calculations). Supplementary Figure 1 replots data from Figure 6, including only retinas for BSS/PBS and Bead/PBS groups with responses within the 99% confidence intervals. From these traces, increased amplitude of responses in the Bead/NLY compared to the Bead/PBS group (including Off-responses) can be more readily observed.

Although effects observed in MEA experiments were in agreement with suggested improvement of retinal function in the NLY01 treated group, this difference was not significant, and a larger number of recordings are needed to definitively determine functional effects.

## Discussion

A growing body of evidence suggests that GLP-1R agonists may be protective in multiple neurodegenerative diseases including glaucoma (Bertilsson et al., 2008; Harkavyi et al., 2008; Kim et al., 2009; Li et al., 2009; Erbil et al., 2019; Park et al., 2021; Sterling et al., 2021). This study investigated neuroprotective effects of several commercially available GLP-1R agonists in a hypertensive mouse model of glaucoma. Results demonstrated RGC protection, reduced microglia/macrophage activation, and variable reduction of ON axon degeneration in response to topical and systemic GLP-1R agonist administration.

The various GLP-1R agonists possess different properties. Liraglutide is an acetylated form of GLP-1 that binds to albumin, resulting in enhanced half-life in both humans and mice (13 and 23 h, respectively). Lixisenatide is a shorter-acting GLP-1 mimetic with a half-life of approximately 3 h in humans (Elkinson and Keating, 2013). Both drugs have shown neuroprotective effects in animal models of Alzheimer’s disease and diabetic retinopathy (McClean et al., 2011; McClean and Hölscher, 2014 Hernández et al., 2016). In comparison, NLY01 is a GLP-1R agonist with a long half-life in humans (12.5 days) and mice (38 h.), and effectively penetrates the blood-brain barrier (Gharagozloo et al., 2021). In a mouse model of oxygen-induced retinopathy, intravitreal NLY01 inhibited microglia/macrophage activation, decreased the number of myeloid cells in the retina, and reduced retinal neovascularization secondary to ischemia (Zhou et al., 2021). In mouse models of Parkinson’s and Alzheimer’s disease, NLY01 prevented neurodegeneration and improved behavioral deficits, learning, and memory (Yun et al., 2018; Park et al., 2021). NLY01 was also shown to reduce the release of IL-1α, TNF-α, and C1q by microglia *in vitro* (Yun et al., 2018). A phase II clinical trial examining the safety and efficacy of NLY01 in treating patients with Parkinson’s is ongoing.

In this study, topically administered liraglutide and lixisenatide resulted in incomplete rescue of RGCs in a mouse model of hypertensive glaucoma, while systemically administered liraglutide and NLY01 were neuroprotective to a greater extent, demonstrating near complete rescue of RGCs. The presence of neuroprotection in the topical group argues for the presence of GLP-1 receptors in the eye itself, thereby allowing GLP-1R agonists to act locally. The relative suppression of *Il1a*, *Tnf*, and *C1qa* mRNA expression in retinal macrophage/microglia suggests that mechanisms of action for other GLP-1R agonists are similar to what we have previously shown for NLY01 (Sterling et al., 2020). Because we did not evaluate ocular concentrations of GLP-1R agonists following topical administration, it is unknown whether improved protection might result from optimization of drug delivery. The greater degree of neuroprotection observed in the systemic arm suggests mechanistic differences between systemic and topical administration. Because changes in glucose homeostasis also impact neuronal survival, it is conceivable that GLP-1R agonists’ incretin effects are in part responsible for neuroprotection in the systemic group (Bu et al., 2013; Song et al., 2016; Agostinone et al., 2018; Al Hussein Al Awamlh et al., 2021; Enz et al., 2021).

Intriguingly, compared to the other bead-injected groups, lower IOP was shown following 2 weeks of treatment with topical liraglutide. This was not observed following topical lixisenatide treatment, nor following systemic liraglutide or NLY01 treatments. This raises the interesting question of whether topical liraglutide might directly lower IOP. GLP-1R agonists have been shown to inhibit Na + K + ATPase-dependent secretion of cerebrospinal fluid at the choroid plexus (Botfield et al., 2017), and the GLP-1R agonist exenatide has shown preliminary effectiveness for treating intracranial hypertension through this mechanism (Mitchell et al., 2023). Because aqueous humor secretion is similarly dependent upon the action of the Na + K + ATPase, inhibition of active secretion could be a pathway through which GLP-1R agonists directly lower IOP. Overall, however, further studies are needed to fully explore this interesting finding, as the status of GLP-1 receptor expression in the eye remains largely unknown, and it does not explain why topical lixisenatide did not similarly result in IOP lowering.

While the number of Iba1 + myeloid cells did not significantly differ between GLP-1R-treated and untreated groups, a trend toward a greater number of myeloid cells in bead-injected vs. BSS-injected eyes was observed [7 ± 0.2 (SEM) vs. 6 ± 0.3 cells per high power field [HPF], respectively]. While it is conceivable that the scarcity of Iba1 + cells per HPF contributed to the lack of statistical significance, it is also possible that cytokine expression and cellular activation states, as opposed to the number of myeloid cells in the retina, are more salient in glaucoma pathogenesis. While NLY01 has previously been shown to reduce microglia density in a mouse model of Parkinson’s, the quantification method used was notably different from that used in our study (Yun et al., 2018). In that study, instead of masked individuals manually counting the number of Iba1 + cell somas, imageJ was used to quantify relative Iba1 intensity which herald activation states.

The effect of GLP-1R agonist administration on ON axon survival in ocular hypertension was more variable. While topical administration of lixisenatide demonstrated significant axon preservation, effects of topical liraglutide did not demonstrate significance. Similarly, systemic treatment with liraglutide significantly rescued ON axons, while effects of NLY01 failed to reach significance despite trending in that direction. ON axon quantification was automated using the Axonet plug-in for FIJI, which may have resulted in greater variability compared to manual counting (Ritch et al., 2020). Further, the wide natural variation in ON axon numbers in mice might have served to obscure relatively small protective effects (Williams et al., 1996). Although our sampling and analysis techniques, which used the fellow-eye as control where possible to highlight treatment-associated differences in neuronal survival that otherwise would have been masked by inter-subject variability (Curcio and Allen, 1990; Williams et al., 1996), have been verified through multiple publications, it is conceivable that our choice of techniques contributed to the relatively large variations we observed between fellow eyes. A larger cohort in a follow up study will be necessary for detecting relatively small differences, if present. This may have been a factor in the systemic groups, where the larger number of eyes in the liraglutide group could have contributed to the detection of significance in ON axon rescue.

The MEA measurements of *ex vivo* NLY01-treated and untreated retinas evaluated the functionality of surviving RGCs in the setting of induced ocular hypertension. While the limited N did not allow for evaluation of significance, RGC function in NLY01-treated retinas with elevated IOP were comparable to untreated retinas without IOP elevation. Overall, MEA findings are consistent with results from other tests, suggesting that GLP-1R agonist administration resulted in both structural and functional rescue of RGCs.

Our previous work supports neuroinflammation as a mechanism of glaucomatous damage and demonstrates RGC rescue by the novel GLP-1R agonist NLY01 (Sterling et al., 2020). This paper extends those findings by demonstrating improved RGC survival and decreased neuroinflammation following topical and systemic treatment with other FDA-approved GLP-1R agents. Results further support a role for GLP-1R agonists as a commercially available class of agents with a known safety profile that may provide a supplemental approach to treating glaucoma without relying exclusively on IOP lowering.

## Data availability statement

The raw data supporting the conclusions of this article will be made available by the authors, without undue reservation.

## Ethics statement

This animal study was reviewed and approved by the Institutional Animal Care and Use Committee (IACUC) of the University of Pennsylvania.

## Author contributions

EL, JS, and QC contributed to the conception and design of the study. EL, MG, TS, SN, JW, JL, JS, and QC contributed to the acquisition and analysis of data. EL, SN, and QC contributed to drafting the text and preparing the figures. All authors contributed to the article and approved the submitted version.
