# Supplementary material for: Topical and systemic GLP-1R agonist administration both rescue retinal ganglion cells in hypertensive glaucoma
Source: Front Cell Neurosci. 2023 Jun 9;17:1156829. doi: 10.3389/fncel.2023.1156829 (PMC10288152; doi:10.3389/fncel.2023.1156829)
Supplement: Supplementary file 1 [file Data_Sheet_1.docx]

Supplementary Material

**Topical and Systemic GLP-1R Agonist Administration both Rescue Retinal Ganglion Cells in Hypertensive Glaucoma**

**Emily C.N. Lawrence^1^, Michelle Guo^1^, Turner D. Schwartz^1^, Sergei Nikonov^1^, Jie Wu^1^, Jingwen Lu^1^, Jacob K. Sterling^1^, Qi N. Cui^1^**

**^1^Scheie Eye Institute, Department of Ophthalmology, University of Pennsylvania, Philadelphia, PA, United States**

**Correspondence:**Emily Lawrence
[**lawrence@pennmedicine.upenn.edu**](mailto:lawrence@pennmedicine.upenn.edu) **qicuilab.org**

# Supplementary Figure 1

NLY01 improves assessment of RGC function in hypertensive glaucoma. At 63 days post-ocular injections, small patches of NLY01 treated and control retinas were stimulated with flashes of light for multielectrode array recording. Group-averaged retinal responses to flashes of 3 different intensities were calculated using 100 ms time bin, with each row corresponding to a particular intensity as indicated on the far left of graphs A-C. Blue bar = light stimulation. Two high-performing “Bead/PBS” retinas were excluded from final analysis. Amplitudes of transient On- (D), sustained On- (E), and Off- (F) responses suggest better RGC function following NLY01 treatment for all three measures that is most pronounced at higher flash intensities. N=11 for BSS/PBS, N=8 for Bead/PBS; N=4 for Bead/NLY01, N=6 for BSS/NLY01.

**
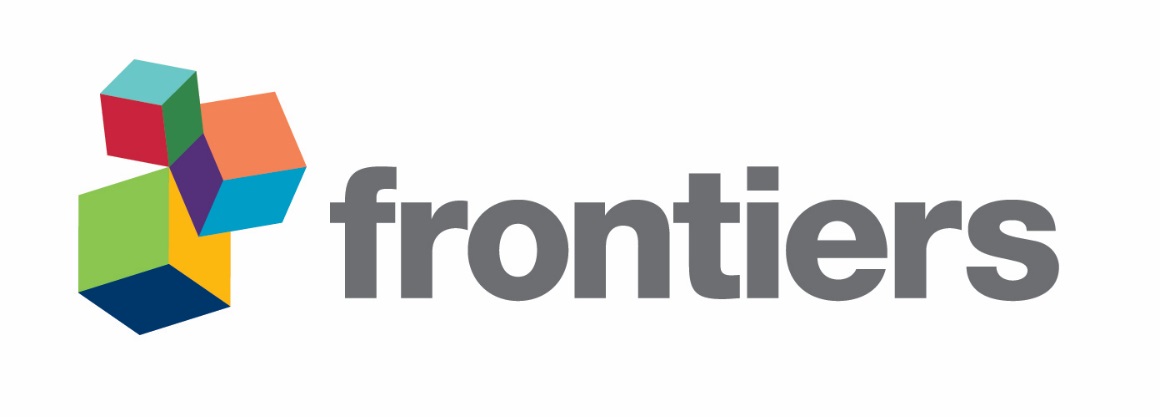
**
