# Supplementary figures and images for: Topical and systemic GLP-1R agonist administration both rescue retinal ganglion cells in hypertensive glaucoma
Source: Front Cell Neurosci. 2023 Jun 9;17:1156829. doi: 10.3389/fncel.2023.1156829 (PMC10288152; doi:10.3389/fncel.2023.1156829)

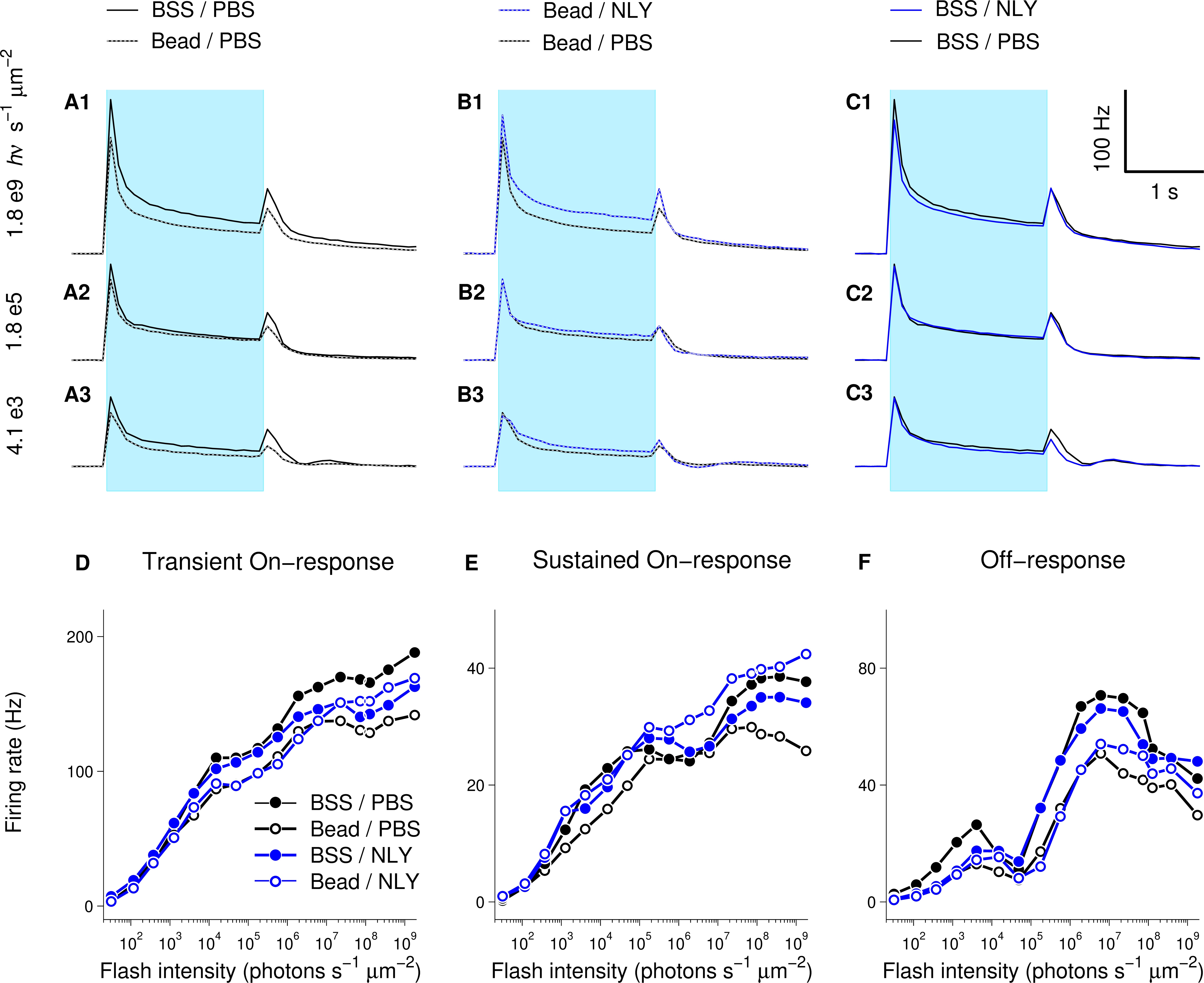

Supplement: Supplementary file 2 [file Image_1.JPEG]
